# Supplementary material for: Evolutionary regulation of human Fas ligand (CD95L) by plasmin in solid cancer immunotherapy
Source: Nat Commun. 2025 Jul 1;16:5748. doi: 10.1038/s41467-025-60990-0 (PMC12217004; doi:10.1038/s41467-025-60990-0)
Supplement: Supplementary file 1 — Supplementary Information [file 41467_2025_60990_MOESM1_ESM.pdf]

SUPPLEMENTARY DATA FIGURES

Title

Evolutionary Regulation of Human Fas Ligand (CD95L) by Plasmin in Solid Cancer Immunotherapy

Author List

Brice E.N. Wamba[1, 2] , Tanmoy Mondal[1, 2], Francis Freenor V [1, 2] , Mehr Shaheed [1, 2, 3], Oliver Pang [1, 2, 3], Daniel Bedinger[4], Patrick Legembre[5], Laurent Devel[6], Sanchita Bhatnagar[2] , Gary Scott Leiserowitz[7], and Jogender Tushir-Singh[1, 2, 7, 8*]

Affiliations

[1]Laboratory of Novel Biologics, University of California Davis, Davis CA 95616, USA
[2]Deartment of Medical Microbiology and Immunology, University of California Davis, Davis CA 95616, USA
[3]Undergraduate Research Volunteers Program, University of California Davis, Davis CA 95616, USA
[4]Carterra, 825 N. 300 W. Ste. C309, Salt Lake City UT 84103, USA
[5]UMR CNRS 7276, INSERM U1262, University of Limoges, Limoges, France
[6]CEA, INRAE, Médicaments et Technologies pour la Santé (MTS), SIMoS, Université Paris-Saclay, 91191, Gif-sur-Yvette, France
[7] Department of Obstetrics and Gynecology, UC Davis School of Medicine, University of California Davis, Davis CA 95616, USA
[8]UC Davis Comprehensive Cancer Center, UC Davis School of Medicine, University of California Davis, Davis CA 95616, USA

*Correspondence: jtsingh@ucdavis.edu

Supplemental Figure 1

a

rs2101810509

Current Build 156
Released September 21, 2022

**Organism**	*Homo sapiens*	**Clinical Significance**	Not Reported in ClinVar
**Position**	chr1:172665627 (GRCh38.p14)	**Gene : Consequence**	FASLG : Missense Variant
**Alleles**	T>C	**Publications**	0 citations
**Variation Type**	SNV Single Nucleotide Variation	**Genomic View**	See rs on genome
**Frequency**	None		

Variant Details | Clinical Significance | HGVS | Submissions | History | Publications | Flanks

Genomic Placements

Sequence name	Change
FASLG RefSeqGene (LRG_58)	NG_007269.1:g.11583T>C
GRCh37.p13 chr 1	NC_000001.10:g.172634767T>C
GRCh38.p14 chr 1	NC_000001.11:g.172665627T>C

Gene: FASLG, Fas ligand (plus strand)

Molecule type	Change	Amino acid[Codon]	SO Term
FASLG transcript variant 1	NM_000639.3:c.457T>C	S [TCC] > P [CCC]	Coding Sequence Variant
FASLG transcript variant 2	NM_001302746.2:c.*27=	N/A	3 Prime UTR Variant
tumor necrosis factor ligand superfamily member 6 isoform 1	NP_000630.1:p.Ser153Pro	S (Ser) > P (Pro)	Missense Variant

b

Source: Genome Aggregation Database (GnomAD)

T= 172665627T
C=172665628C
C=172665629C
] TCC = Ser

C=172665627C
C=172665628C
C=172665629C
] CCC = Pro

c

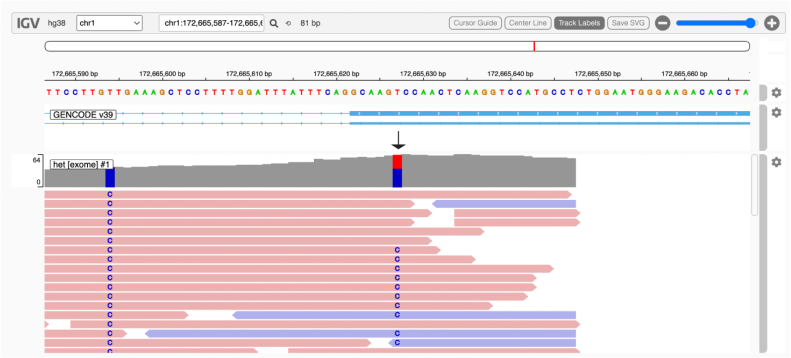

d

Genetic Ancestry Group Frequencies

Genetic Ancestry Group	Allele Count	Allele Number	Number of Homozygotes	Allele Frequency
South Asian	1	91080	0	0.00001098
African/African American	0	75054	0	0.000
Admixed American	0	60024	0	0.000
Ashkenazi Jewish	0	29606	0	0.000
East Asian	0	44886	0	0.000
European (Finnish)	0	62884	0	0.000
Middle Eastern	0	6062	0	0.000
European (non-Finnish)	0	1180026	0	0.000
Amish	0	912	0	0.000
Remaining	0	62496	0	0.000
**Total**	**1**	**1613030**	**0**	**6.200e-7**

Supplementary Fig. 1 a Single nucleotide variation (SNV: rs2101810509) details at position of chr1:172665627 (GRCh38.p14). Alleles T>C results into Ser to Pro mutation. **b** The exact position on chr1 (172665627, 172665628, 172665629) produces a TCC codon for serine and single nucleotide variation at 172665627 from T to C results in a codon CCC for proline. **c** Interactive IGV.js visualization showing reads for the variant rs2101810509. Reads may not be present for every sample carrying this variant. **d** Genetic ancestry group frequencies showing the detected rare variant in the South Asian population, Allele Count:1, Allele Number:1460718, Allele Frequency: 6.846e-7

Supplemental Figure 2

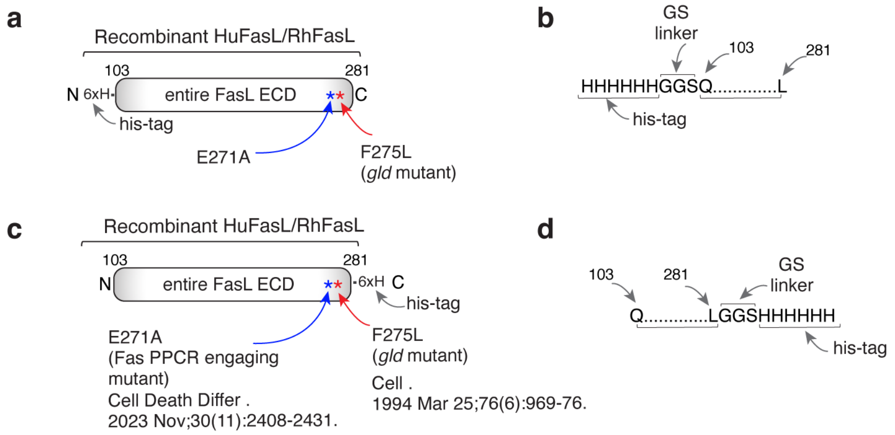

e

CHO expression	N-termial His	C-termial His
Yield/100 ml culture (1)	0.55mg/ml (1.0ml)	0.18mg/ml (1.0ml)
Yield/200 ml culture (2)	0.80mg/ml (1.5ml)	0.31mg/ml (1.3ml)
Yield/300 ml culture (3)	1.25mg/ml (1.6ml)	0.48mg/ml (1.2ml)
Yield/500 ml culture (4)	1.95mg/ml (2.0ml)	0.65mg/ml (1.0ml)

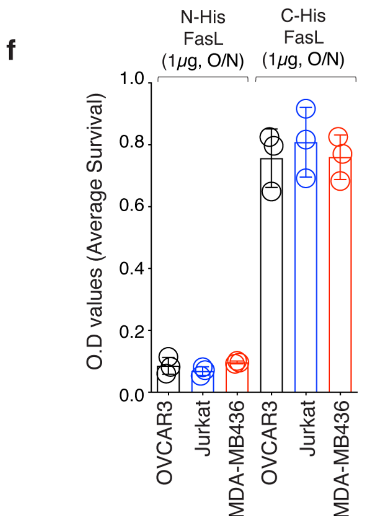

Supplementary Fig. 2 a, b Schematic detail depiction of N-terminal his-tagged HuFasL (103-281aa) and RhFasL (103-281aa) generation. The his-tag and FasL sequences are separated with three amino acid GGS linkers. A blue and red asterisk indicates E271A and F275L FasL mutations described in the literature. **c, d** Schematic detail depiction of C-terminal his-tagged HuFasL (103-281aa) and RhFasL (103-281aa) generation. The FasL and his-tag are separated with three amino acid GGS linkers. A blue and red asterisk indicates E271A and F275L FasL mutations described in the literature. **e** CHO cell expression data of N-terminal his-tagged and C-terminal his-tagged human FasL. **f** Cell survival assay of indicated cell lines treated with N-terminal his-tagged and C-terminal his-tagged of human FasL. Error bars in (**f**) represent SD (n=3).

Supplemental Figure 3

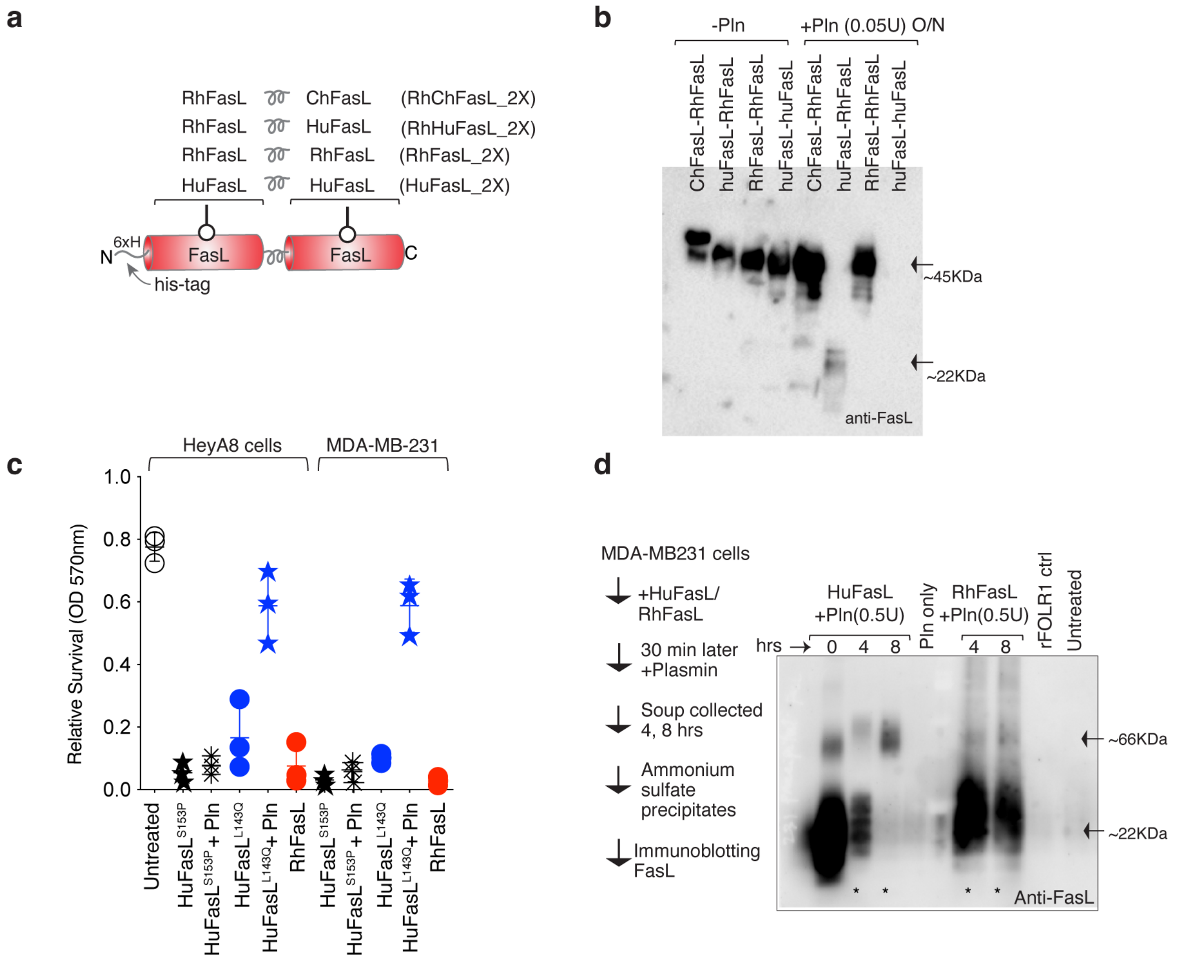

Supplementary Fig. 3 a Schematic of genetic construction of various combinations of human, rhesus, and chimpanzee FasL to generate his-tagged 2XFasL constructs. **b** The proteins generated (as in **a**) were incubated with ± plasmin followed by SDS-PAGE and immunoblotting using anti-FasL antibody. **c** Survival analysis of indicated cell lines treated with indicated FasL proteins ± plasmin (Pln). **d** The MDA-MB231 cells were treated with huFasL and RhFasL, followed by plasmin treated for the indicated time. The culture supernatant was precipitated using ammonium sulfate, followed by immunoblotting for FasL. Error bars in (**c**) represent SD (n=3).

Supplemental Figure 4

a

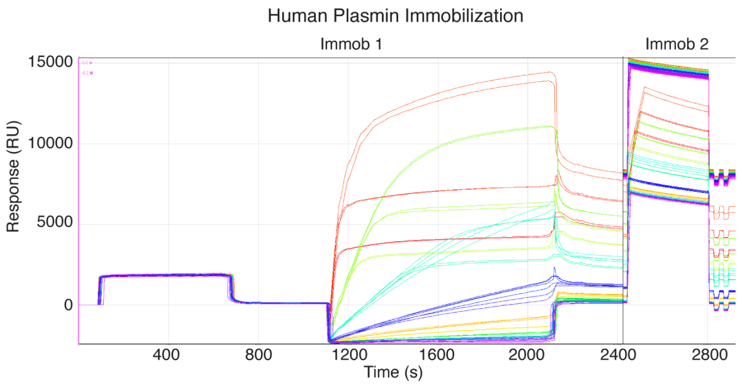

b

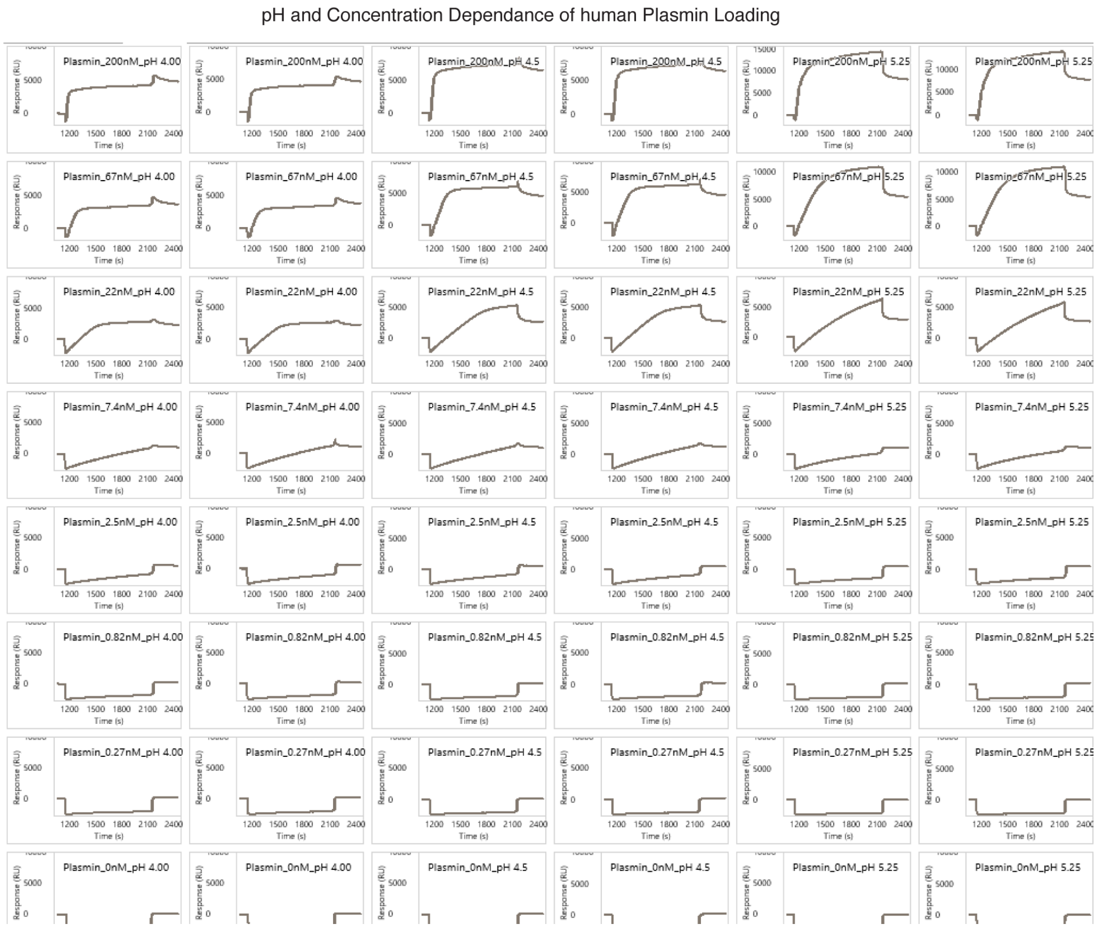

Supplementary Fig. 4 a The pH and concentration dependence of human plasmin loading. The human plasmin was covalently linked to an HC30M (30 nM linear polycarboxylate) chip surface in the presence of a coupling running buffer of 30 mM MES pH 5.5 + 130 mM NaCl and 0.05% Tween 20, followed by activation with 20 mM EDC + 5 mM S-NHS in 100 mM MES pH 5.5 for 7 minutes. **b** Histograms showing human plasmin titration series prepared at indicated pH values in duplicate. The pH 5.25 yielded the highest immobilization levels.

Supplemental Figure 5

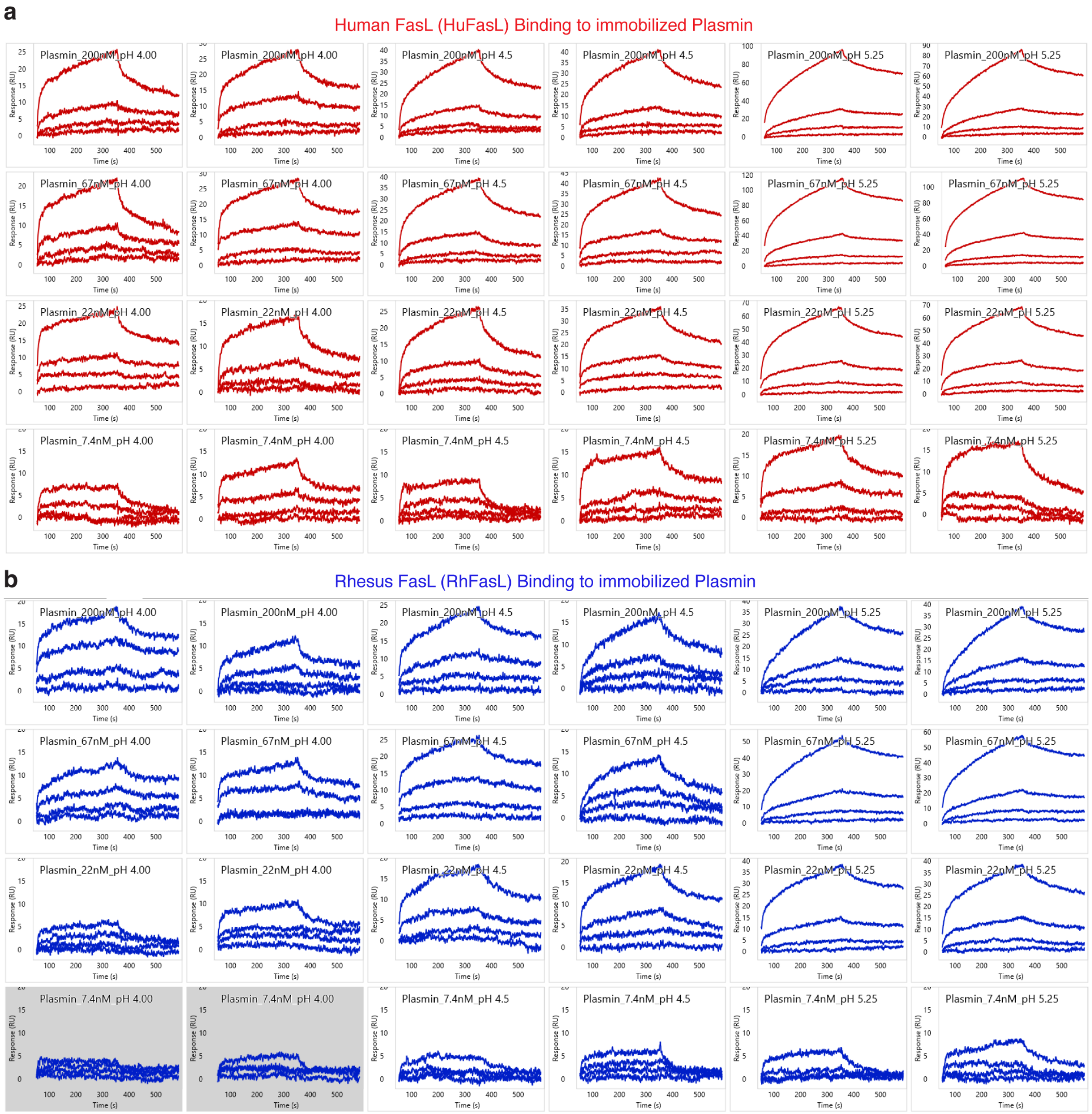

Supplementary Fig. 5 a, b Histogram showing kinetic injections and binding behavior of human (a) and monkey (b) FasL with the increasing concentration of immobilized plasmin at indicated pH values.

Supplemental Figure 6

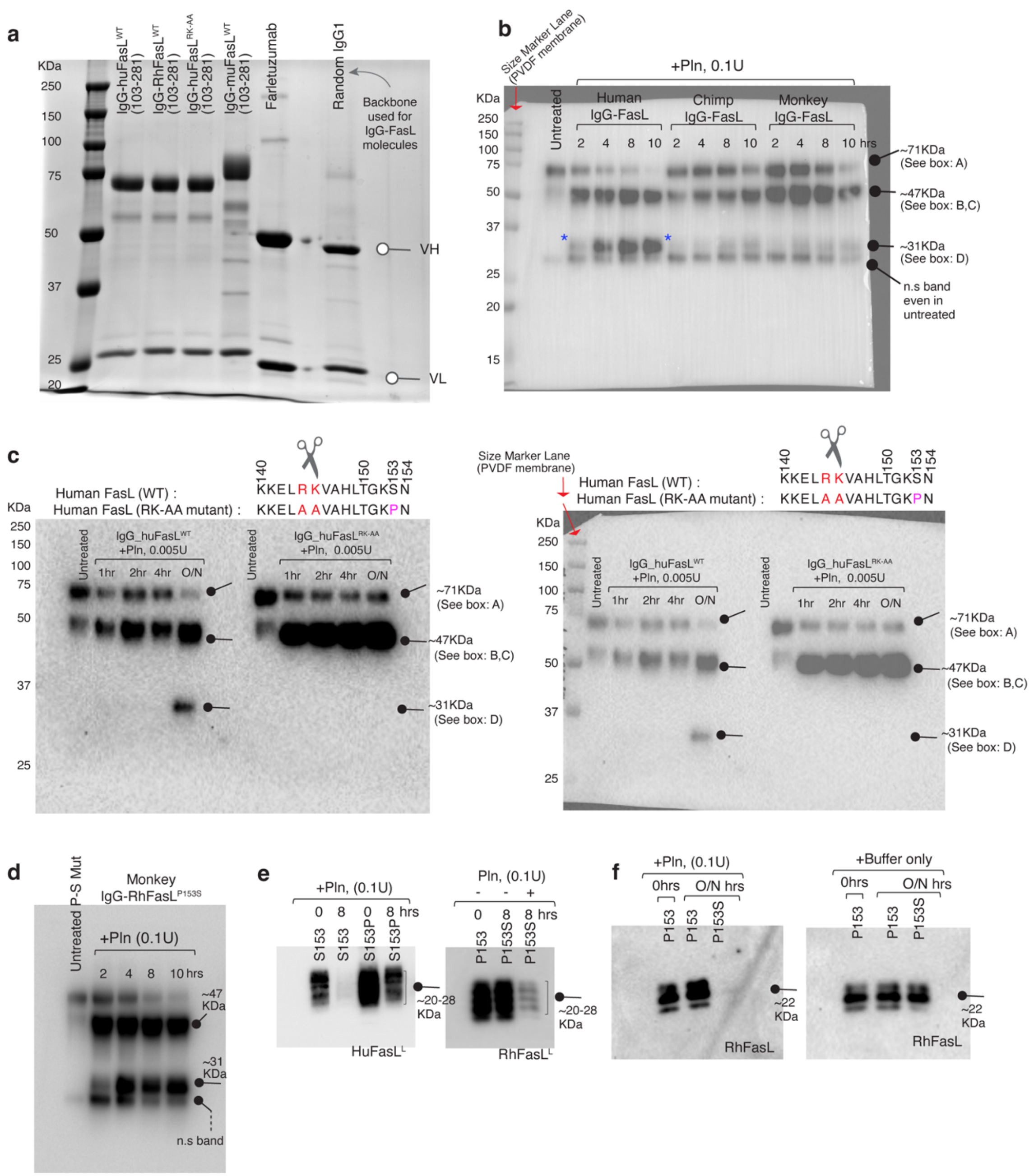

Supplementary Fig. 6 a Indicated random-IgG1-conjugated FasL molecules were run on the SDS page in reducing condition following the staining with the Coomassie blue dye. Additional anti-FOLR1 and Random IgG molecules were run for size control, which showed reduced ~50KDa heavy chain (VH) and ~25KDa light chain (VL). The random-IgG1-conjugated FasL molecules had the same VL size, but the VH was larger, around 71KDa. The VH in IgG1-muFasL is considerably larger in size than IgG1-huFasL potential due to the additional N-linked glycosylation site (See sequence in Fig 4a). **b, c** Same Fig 3c and Fig 3d image except the first lane loaded with precision plus protein dual color standard marker (1610374) on PVDF membrane was imaged with colorimetric detection method to capture pre-defined ladder sizes as indicated. **d** Same as Fig 3c and Fig 3d except rhesus IgG-FasL with P153S mutation was used in the time course Pln cleavage experiment. **e** Indicated his-tagged HuFasL (top) and his-tagged RhFasL (bottom) with indicated mutations at the top, which were incubated with Pln followed by immunoblotting with FasL. **f** Indicated his-tagged RhFasL with indicated mutations at the top, which were incubated with Pln overnight, followed by immunoblotting with FasL.

Supplemental Figure 7

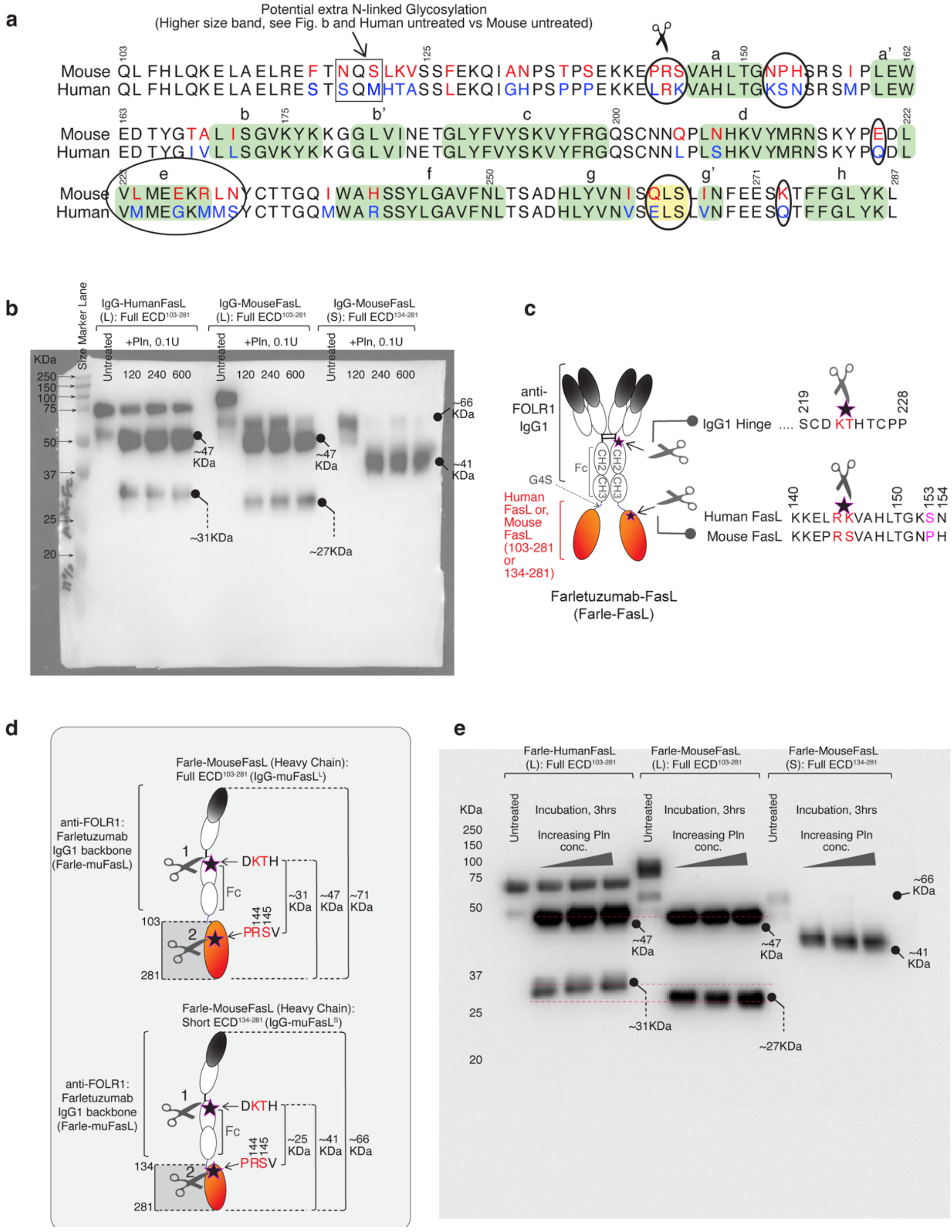

Supplementary Fig. 7 a Sequence alignment of human and mouse FasL entire ectodomain. The arrow indicates potentially extra N-linked glycosylation sites in mouse FasL. The scissor indicates the dissimilar sequence near the ^{144}RK^{145} plasmin cleavage site. **b** Same Fig. 4f image, except the first lane loaded with precision plus protein dual color standard marker (1610374) on PVDF membrane was imaged with a colorimetric detection method to capture pre-defined ladder sizes as indicated. **c** Same as Fig 3a and b, except instead of random IgG1, anti-FOLR1 (Farletuzumab) was used to engineer IgG conjugated human and murine FasL, as shown in the schematic. **d** Schematic and size comparison of Farle-MuFasL^{L} and Farle-MuFasL^{S}. Similar to Fig.4 d and e, the potential fragment release is indicated with the exact sizes in KDa. **e** Same Fig. 4f except that instead of random IgG1, anti-FOLR1 conjugated FasL forms (of human and mouse) were used in plasmin cleavage assay.

Supplemental Figure 8

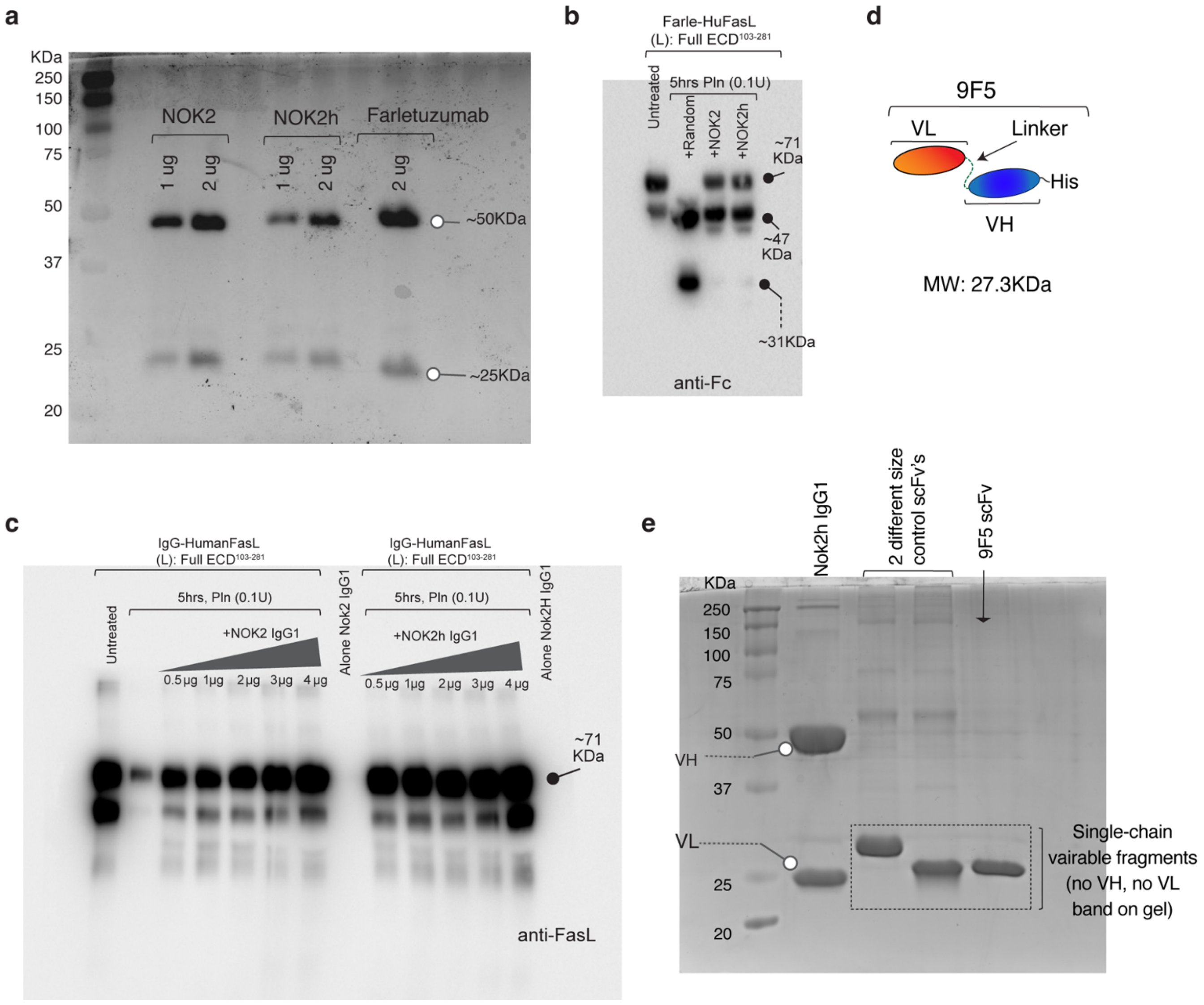

Supplementary Fig. 8 a Cloned and purified Nok2 and Nok2h IgG2a (along with Farletuzumab IgG1) run on reducing gel to confirm the sizes after staining with the Coomassie blue dye. **b** In-vitro Farle-huFasL cleavage assay as in Fig 5b in the presence of preincubated with Nok2 and Nok2h IgG2a antibodies (37°C, 1 hr) before adding recombinant plasmin. Following the assay, samples were run on SDS-PAGE followed by immunoblotting using anti-Fc antibody. **c** Random-IgG1-conjugated huFasL were incubated with the increasing concentration of Nok2 and Nok2h IgG2a antibodies (37°C, 1 hr), followed by plasmin addition for 5 hrs at 37°C. Following the assay, the samples were run on SDS-PAGE followed by immunoblotting using anti-FasL (not anti-Fc) antibody. **d** Schematic of 9F5 scFv genetic construction. **e** Cloned and purified 9F5 scFv using Ni-NTA his-resin beads was run on reducing gel (along with other scFvs and IgG1) to confirm the sizes using Coomassie blue dye stain.

Supplemental Figure 9

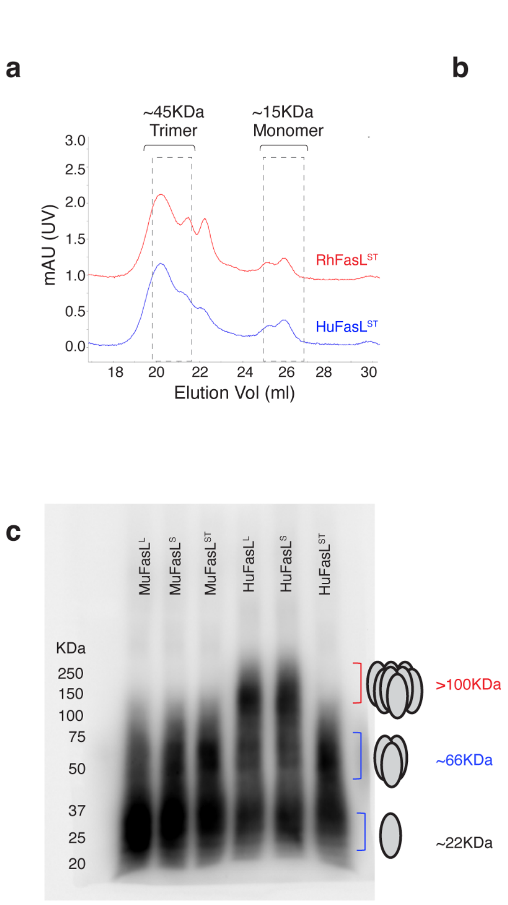

Supplementary Fig. 9 a SEC chromatograms of indicated human and rhesus FasL (plasmin cleaved form: HuFasLST, RhFasLST) are shown with different colors. **b** The engineered Nok2h-muFasL^{L} and Nok2h-huFasL^{L}, as indicated (along with Nok2h IgG1) as in Fig 6e, were purified using protein-A resin and run on reducing gel to confirm the sizes by staining with the Coomassie blue dye. **c** Indicated forms of his-tagged human and murine FasL were added onto OVCAR3 cells for 2 hours in serum-free media followed by precipitation of supernatant. The samples were loaded (without boiling) onto non-reducing and partly denaturing gels to capture the potential aggregates by FasL immunoblotting.

Supplemental Figure 10

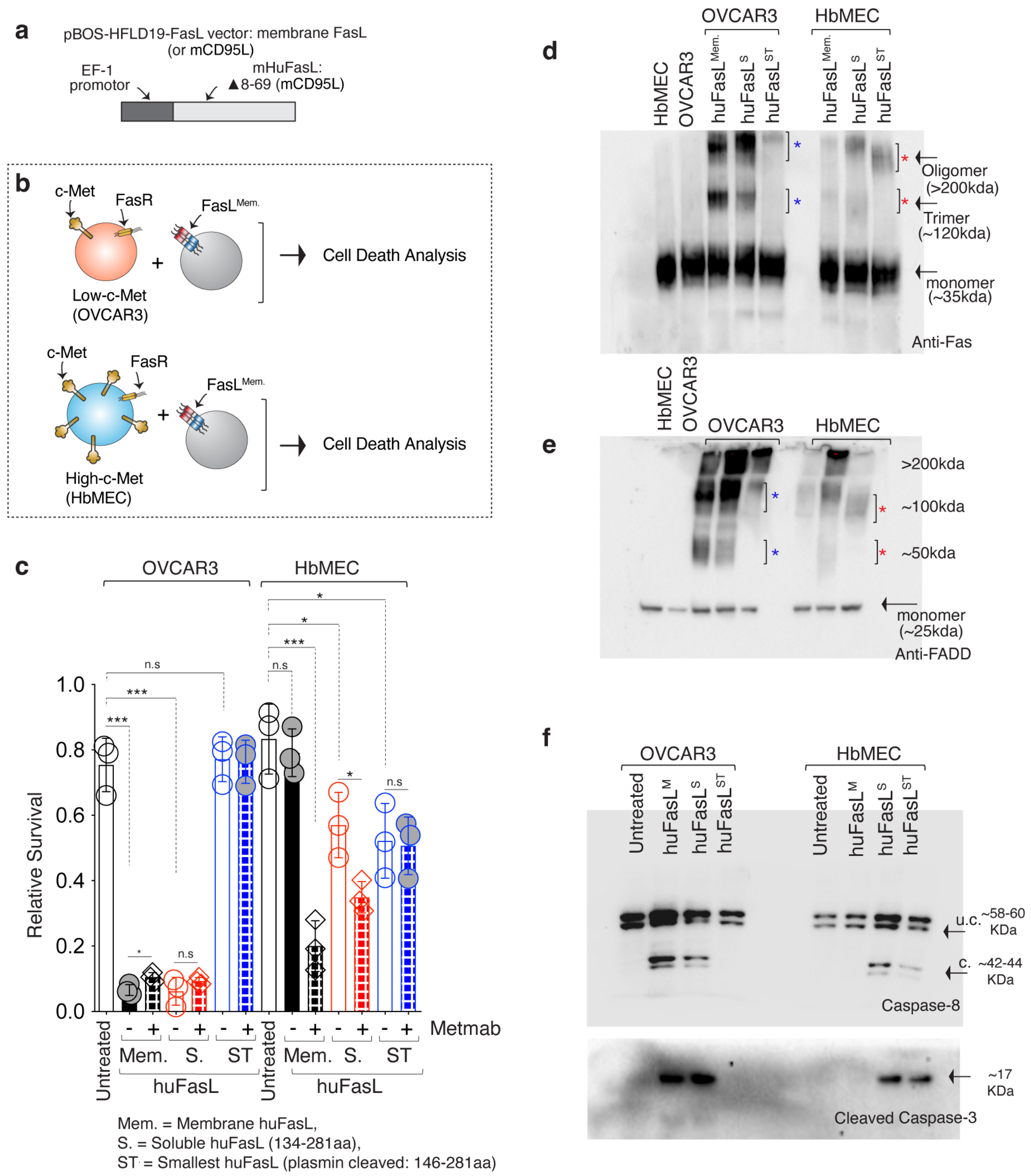

Supplementary Fig. 10 a The Schematic of pBOS-HFLD19-FasL vector to transiently express membrane FasL (or mCD95L). We consistently observed 40-50% CHO-K cells expressing membrane FasL (FasL^{Mem}), see also Fig 7i or extended Fig 11e). **b** Schematic showing mixing experiments of transient membrane FasL (FasL^{Mem}) expressing CHO-K cells with either low c-Met expressing OVCAR3 or high c-Met expressing HbMEC cells. **c** In one case, the FasL^{Mem} expressing CHO-K cells were mixed with OVCAR3 and HbMEC cells for 24 hrs, followed by cell death analysis. In another case, empty vectors expressing CHO-K cells were mixed with OVCAR3 and HbMEC cells and indicated 1μg soluble huFasL (huFasL^{S}: 134-281aa or huFasLST:146-281aa) was added for 24 hrs followed by cell death MTT assays. Another full set of same experiments was carried out in exactly similar condition in presence of anti-c-Met antibody 21μg Metmab (n=3). OVCAR3: Untreated vs. Mem. huFasL, ***p= 0.0001; Mem. huFasL vs. Mem. huFasL + Metmab, *p= 0.0339; Untreated vs. S. huFasL, ***p= 0.0002; S. huFasL vs S. huFasL + Metmab, nsp= 0.2830; Untreated vs. ST huFasL, nsp= 0.7924; ST huFasL vs ST huFasL + Metmab, nsp=0.8958; HbMEC: Untreated vs. Mem. huFasL, nsp= 0.6036; Mem. huFasL vs. Mem. huFasL +

Metmab, ***p= 0.0006; Untreated vs. S. huFasL, *p= 0.0358; S. huFasL vs S. huFasL + Metmab, *p = 0.0260; Untreated vs. ST huFasL, *p= 0.0263; ST huFasL vs. ST huFasL + Metmab, nsp= 0.8631; Unpaired, two-sided parametric t-test with no adjustments. **d, e** Same as c, except OVCAR-3 and HbMEC cell lysates after the indicated mixing FasL treatments (top) for 2hrs, were analyzed using non-reducing partly denaturing clustering assays using Fas (d) and FADD (e) immunoblotting. **(f)** Same as d and e, except the total caspase-8 and cleaved caspase-3 profile was analyzed using immunoblotting. u.c. indicates un-cleaved, and c. indicates cleaved caspase-8. Error bars in (**c**) represent SD (n=3).

Supplemental Figure 11

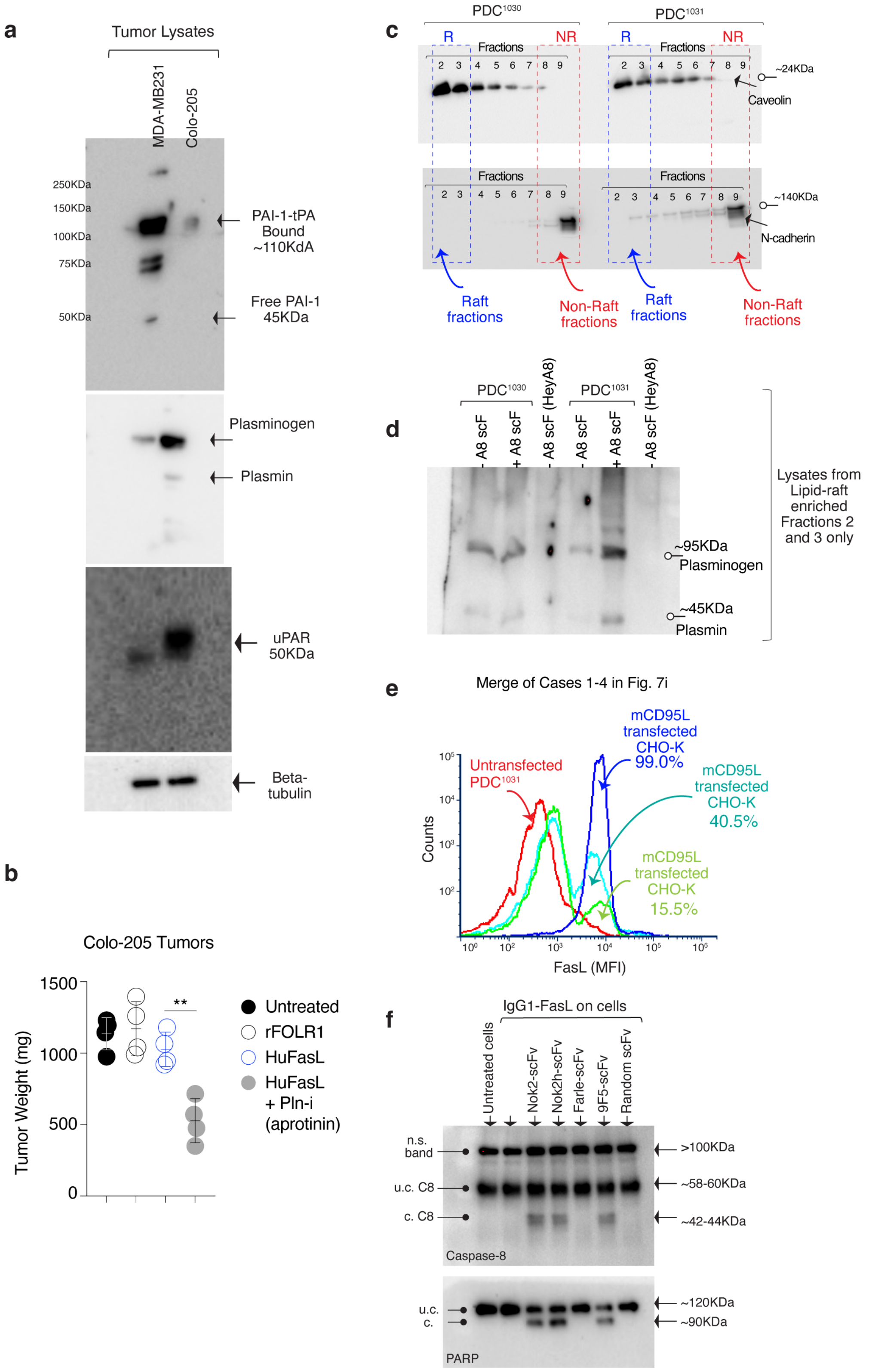

Supplementary Fig. 11 a 8-week-old NSG mice were allowed to form SQ tumors with colo205 and MDA-MB231 cells. Tumors were isolated, and lysates were compared for PAI-1, uPAR, FasR, and plasminogen/plasmin expression using SDS-PAGE and immunoblotting. **b** 8-week-old mice bearing SQ tumors of colo205 cells were i.p. injected with 50µg of indicated FasL alone or ± plasmin inhibitor aprotinin 200nM every alternate day (n = 4). After 4 weeks, isolated tumor weights were quantified. huFasL vs huFasL + Pln-i, **p= 0.0022; Unpaired, two-

sided parametric t-test with no adjustments. **c** To enrich the lipid raft, fractions of indicated cell lines were subjected to triton x-100 solubilization. After the sucrose gradient centrifugations, various fractions (1-10) were run on SDS-PAGE and immunoblotted with caveolin-1(lipid raft enrich maker) and N-cadherin (non-raft marker). **d** Same as c except the raft fractions (of indicated cell lines) were enriched after 6 hrs incubation of the cells with ± 1mg uPA agonist antibody as indicated. The fractions 2 and 3 were combined and run on SDS-PAGE followed by immunoblotting with plasmin antibody. **e** Same at Fig. 7i, except the overlay of 4 data sets is shown from the Ovarian PDC1031 cells and transient membrane huFasL expressing CHO-K cells mixing flow cytometry experiment. **f** IgG-HuFasL was added to confluent PDC1031 cells in the presence of indicated scFvs and controls. After 2 hrs, the supernatant was removed and concentrated using ammonium sulfate for the experiment described in Fig 7k. The left-over cells were added with new media and incubated for an additional 2 hrs, and lysates were immunoblotted for caspase-8 and PARP. In (**f**) u.c. indicates uncleaved, c. indicates cleaved.
Error bars in (**b**) represent SD (n=3).

Extended Figure 12

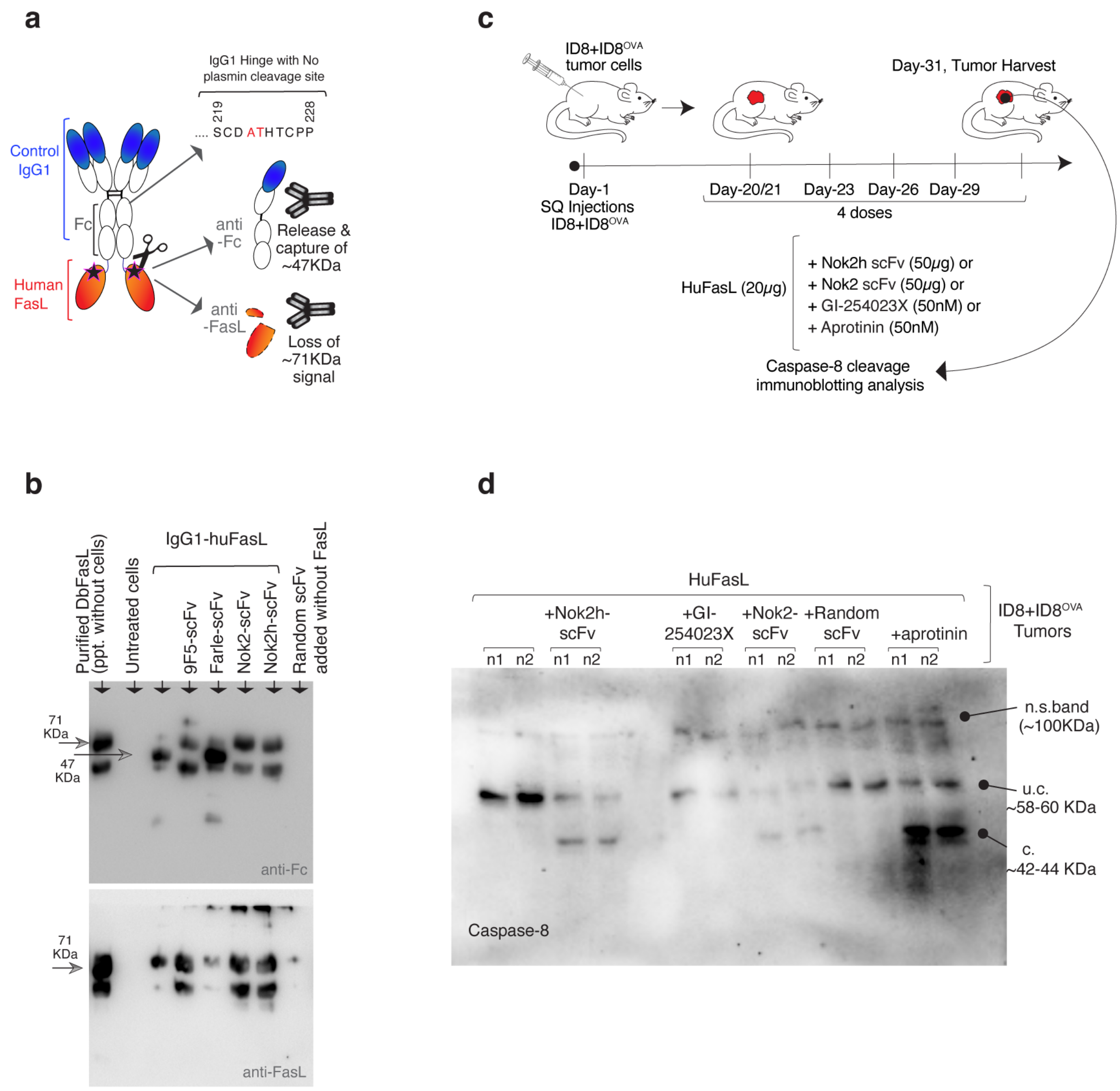

Supplementary Fig. 12 a, b Same as Fig 7j, k except instead of PDC[1031], ID8[OVA] cells were used. **c** Cartoon schematics and timelines of syngenetic mouse tumor experiments (similar to Figs 8 and 9) performed before caspase-8 analysis. **d** Tumor lysates generated after indicated huFasL treatment in the presence and absence of indicated FasL plasmin interfering conditions of either antibodies or inhibitors. In (**d**) u.c. indicates uncleaved, c. indicates cleaved.

Supplemental Figure 13

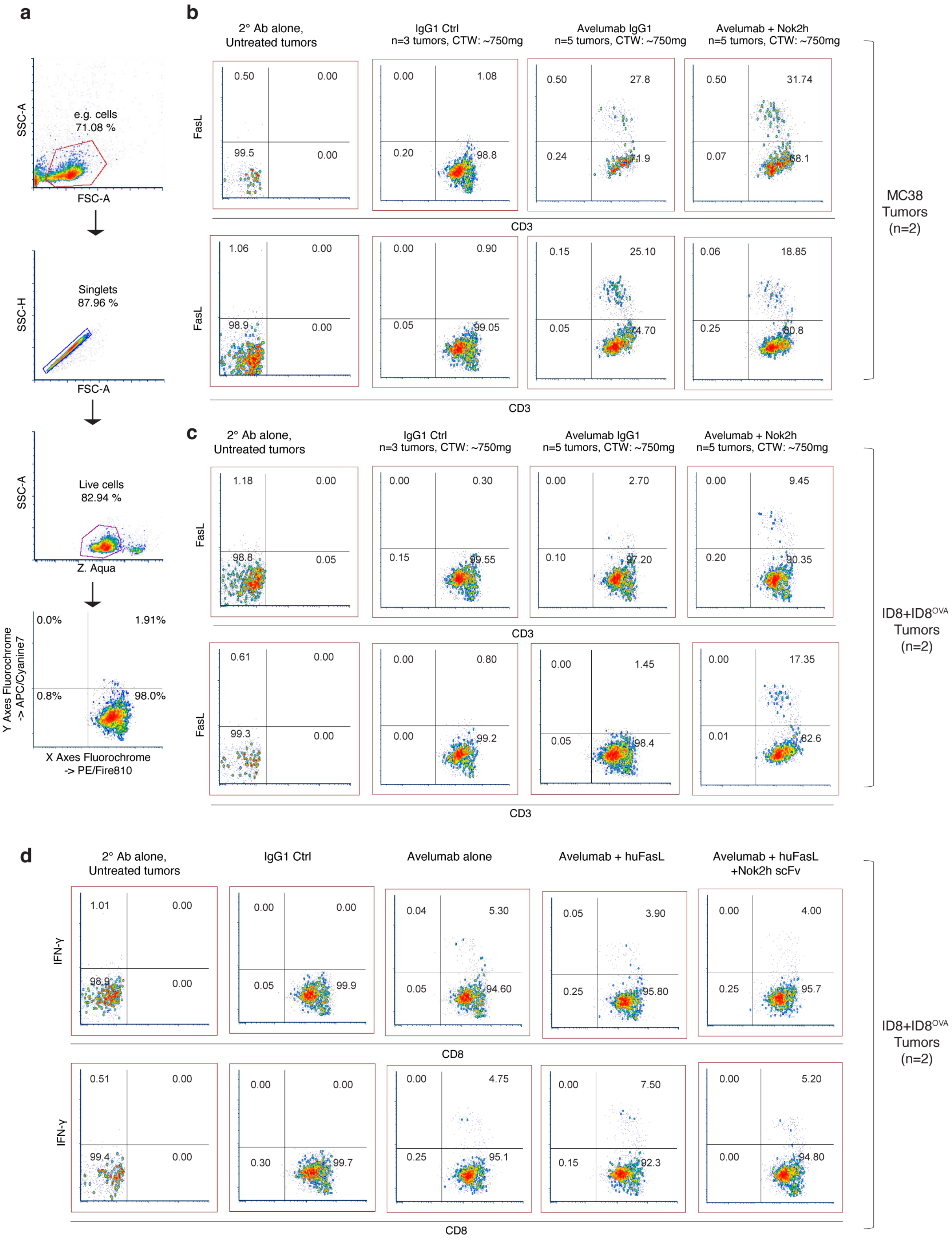

Supplementary Fig. 13 a Gating strategy for data in Fig 8h-k, 9a-c, See methods section for more details. **b, c** Exactly same experiment described in Fig 8h-k, except the data from experiment 1 (n=1) is shown in Fig 8i, j and other two sets (n=2 and n=3) flow chromatogram are shown here for MC38 (a) and ID8^{OVA} tumors (b) after indicated treatments. **d** Same as Fig 9a, b except the data from experiment 1 (n=1) is shown in Fig 9b, and other two sets (n=2 and n=3) flow chromatogram are shown here for the plot data in Fig 9c.

Extended Figure 14

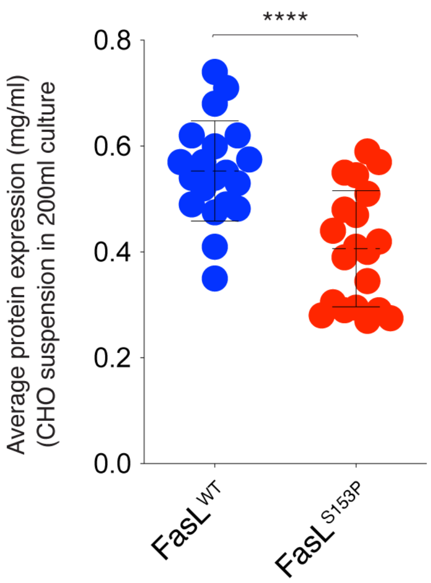

Supplementary Fig. 14 Average protein expression comparison of N-terminal his-tagged FasL^{WTS153} vs FasL^{S153P} in CHO expression system (n >18). huFasL^{WT} vs huFasL^{S153P}, ****p= <0.0001; Unpaired values, two-sided parametric t-test with no adjustments.
