# Supplementary material for: Evolutionary regulation of human Fas ligand (CD95L) by plasmin in solid cancer immunotherapy
Source: Nat Commun. 2025 Jul 1;16:5748. doi: 10.1038/s41467-025-60990-0 (PMC12217004; doi:10.1038/s41467-025-60990-0)
Supplement: Supplementary file 2 — Reporting Summary [file 41467_2025_60990_MOESM2_ESM.pdf]

nature portfolio

Corresponding author(s): Jogender Tushir-Singh

Last updated by author(s): Jun 5, 2025

Reporting Summary

Nature Portfolio wishes to improve the reproducibility of the work that we publish. This form provides structure for consistency and transparency in reporting. For further information on Nature Portfolio policies, see our Editorial Policies and the Editorial Policy Checklist.

Statistics

For all statistical analyses, confirm that the following items are present in the figure legend, table legend, main text, or Methods section.

n/a	Confirmed	
☐	☒	The exact sample size (n) for each experimental group/condition, given as a discrete number and unit of measurement
☒	☐	A statement on whether measurements were taken from distinct samples or whether the same sample was measured repeatedly
☐	☒	The statistical test(s) used AND whether they are one- or two-sided<br>*Only common tests should be described solely by name; describe more complex techniques in the Methods section.*
☒	☐	A description of all covariates tested
☒	☐	A description of any assumptions or corrections, such as tests of normality and adjustment for multiple comparisons
☐	☒	A full description of the statistical parameters including central tendency (e.g. means) or other basic estimates (e.g. regression coefficient) AND variation (e.g. standard deviation) or associated estimates of uncertainty (e.g. confidence intervals)
☒	☐	For null hypothesis testing, the test statistic (e.g. F, t, r) with confidence intervals, effect sizes, degrees of freedom and P value noted<br>*Give P values as exact values whenever suitable.*
☒	☐	For Bayesian analysis, information on the choice of priors and Markov chain Monte Carlo settings
☒	☐	For hierarchical and complex designs, identification of the appropriate level for tests and full reporting of outcomes
☒	☐	Estimates of effect sizes (e.g. Cohen's d, Pearson's r), indicating how they were calculated

Our web collection on statistics for biologists contains articles on many of the points above.

Software and code

Policy information about availability of computer code

Data collection	No Custom code was used.
Data analysis	No Custom code was used. Structures were generated for previously published PDB files in Chimera without any Chimera code changes

For manuscripts utilizing custom algorithms or software that are central to the research but not yet described in published literature, software must be made available to editors and reviewers. We strongly encourage code deposition in a community repository (e.g. GitHub). See the Nature Portfolio guidelines for submitting code & software for further information.

Data

Policy information about availability of data

All manuscripts must include a data availability statement. This statement should provide the following information, where applicable:

- Accession codes, unique identifiers, or web links for publicly available datasets
- A description of any restrictions on data availability
- For clinical datasets or third party data, please ensure that the statement adheres to our policy

Provide your data availability statement here.

Research involving human participants, their data, or biological material

Policy information about studies with human participants or human data. See also policy information about sex, gender (identity/presentation), and sexual orientation and race, ethnicity and racism.

Reporting on sex and gender	This is a mechanistic study for regulation by Plasmin, hence, animals from different sex were randomly selected and both male and females animals were almost equal in numbers for studies.
Reporting on race, ethnicity, or other socially relevant groupings	Not Not Applicable
Population characteristics	Not Applicable
Recruitment	Not Applicable
Ethics oversight	No human samples were used.

Note that full information on the approval of the study protocol must also be provided in the manuscript.

Field-specific reporting

Please select the one below that is the best fit for your research. If you are not sure, read the appropriate sections before making your selection.

[x] Life sciences [ ] Behavioural & social sciences [ ] Ecological, evolutionary & environmental sciences

For a reference copy of the document with all sections, see nature.com/documents/nr-reporting-summary-flat.pdf

Life sciences study design

All studies must disclose on these points even when the disclosure is negative.

Sample size	Most the cases each experiment was repeated n=3 at least.
Data exclusions	Not Applicable
Replication	Biological replicates performed.
Randomization	For in vivo studies mice were randomly selected from equitable population of males and females.
Blinding	Not Applicable

Reporting for specific materials, systems and methods

We require information from authors about some types of materials, experimental systems and methods used in many studies. Here, indicate whether each material, system or method listed is relevant to your study. If you are not sure if a list item applies to your research, read the appropriate section before selecting a response.

Materials & experimental systems

n/a	Involved in the study	
[ ]	[x]	Antibodies
[ ]	[x]	Eukaryotic cell lines
[x]	[ ]	Palaeontology and archaeology
[ ]	[x]	Animals and other organisms
[x]	[ ]	Clinical data
[x]	[ ]	Dual use research of concern
[x]	[ ]	Plants

Methods

n/a	Involved in the study	
[x]	[ ]	ChIP-seq
[ ]	[x]	Flow cytometry
[x]	[ ]	MRI-based neuroimaging

Antibodies

Antibodies used	All the details are provided in methods section.
Validation	All commercially used antibodies are validated by the suppliers and have been published in numerous studies. The size in western blots is also a confirmation of antibody validation. Wherever indicated antigen-negative cells or lysates etc., were used for additional validation.

Eukaryotic cell lines

Policy information about cell lines and Sex and Gender in Research

Cell line source(s)	Please see method section
Authentication	Please see method section
Mycoplasma contamination	Cells were continuously checked and confirmed to be negative for Mycoplasma contamination prior to initiating experiments
Commonly misidentified lines (See ICLAC register)	Not Applicable

Animals and other research organisms

Policy information about studies involving animals; ARRIVE guidelines recommended for reporting animal research, and Sex and Gender in Research

Laboratory animals	Please see method section for the details
Wild animals	No wild animal was used.
Reporting on sex	No findings does not apply to one sex or another. Equal number of both male and female mice were randomly used in the study.
Field-collected samples	No samples were collected form the field.
Ethics oversight	No Ethical approval was required for this mechanistic and biochemical study

Note that full information on the approval of the study protocol must also be provided in the manuscript.

Plants

Seed stocks	Not Applicable
Novel plant genotypes	Not Applicable
Authentication	Not Applicable

Flow Cytometry

Plots

Confirm that:

- [x] The axis labels state the marker and fluorochrome used (e.g. CD4-FITC).
- [x] The axis scales are clearly visible. Include numbers along axes only for bottom left plot of group (a 'group' is an analysis of identical markers).
- [x] All plots are contour plots with outliers or pseudocolor plots.
- [x] A numerical value for number of cells or percentage (with statistics) is provided.

Methodology

Sample preparation	Please see method section
Instrument	Please see method section
Software	Please see method section
Cell population abundance	Please see method section

Gating strategy | Please see method section

☒ Tick this box to confirm that a figure exemplifying the gating strategy is provided in the Supplementary Information.
